# Regulation of Autocrine Signaling in Subsets of Sympathetic Neurons Has Regional Effects on Tissue Innervation

**DOI:** 10.1016/j.celrep.2015.02.016

**Published:** 2015-03-05

**Authors:** Thomas G. McWilliams, Laura Howard, Sean Wyatt, Alun M. Davies

**Affiliations:** 1 Division of Molecular Biosciences, School of Biosciences, Cardiff University, Museum Avenue, Cardiff CF10 3AX, Wales

## Abstract

The regulation of innervation by target-derived factors like nerve growth factor (NGF) is the cornerstone of neurotrophic theory. Whereas autocrine signaling in neurons affecting survival and axon growth has been described, it is difficult to reconcile autocrine signaling with the idea that targets control their innervation. Here, we report that an autocrine signaling loop in developing mouse sympathetic neurons involving CD40L (TNFSF5) and CD40 (TNFRSF5) selectively enhances NGF-promoted axon growth and branching, but not survival, via CD40L reverse signaling. Because NGF negatively regulates CD40L and CD40 expression, this signaling loop operates only in neurons exposed to low levels of NGF. Consequently, the sympathetic innervation density of tissues expressing low NGF is significantly reduced in CD40-deficient mice, whereas the innervation density of tissues expressing high levels of NGF is unaffected. Our findings reveal how differential regulation of autocrine signaling in neurons has region-specific effects on axon growth and tissue innervation.

## Highlights

• CD40/CD40L autocrine signaling enhances NGF-promoted sympathetic axon growth
• NGF negatively regulates CD40 and CD40L levels in developing sympathetic neurons
• Accordingly, CD40/CD40L signaling only enhances axon growth at low levels of NGF
• Innervation of tissues expressing low NGF levels is disrupted in CD40 knockout mice

## Graphical Abstract

**Figure:**
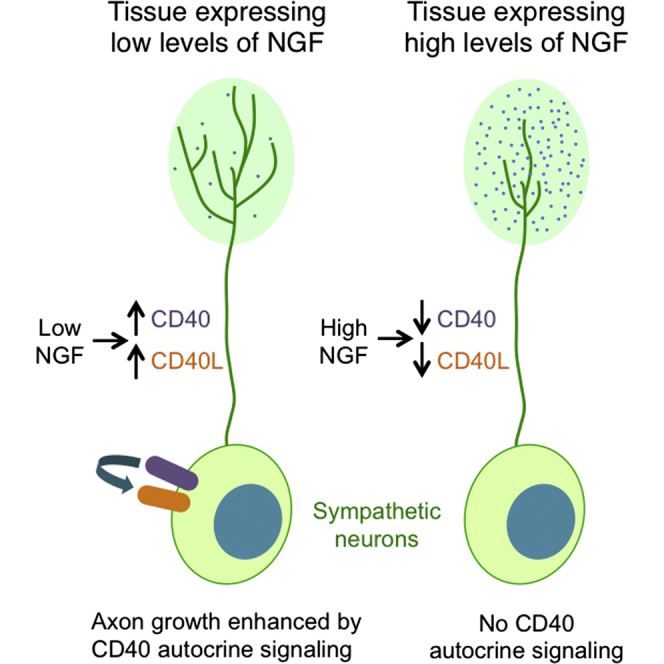

## Teaser

McWilliams et al. report that CD40/CD40L autocrine signaling in developing sympathetic neurons promotes axon growth. Because NGF negatively regulates CD40/CD40L expression, this autocrine-signaling loop operates only in neurons innervating low-NGF-expressing tissues, which are hypoinnervated in CD40 knockout mice. This reveals how autocrine signaling differentially regulates tissue innervation.

## Introduction

Neurotrophic theory provides an explanation for how the target tissues of neuronal populations in the developing peripheral nervous system control their innervation. The basic idea is that tissues synthesize just the right amount of a neurotrophic factor to support the survival of the required number of innervating neurons and promote the growth and branching of their axons within the tissue. Neurotrophic theory is endorsed by a large body of work on nerve growth factor (NGF), the first neurotrophic factor to be identified, and has been corroborated by studies of other members of the NGF family of neurotrophins and by other neurotrophic factors (Levi-Montalcini, 1987, Davies, 2003, Dekkers et al., 2013). In addition to target-derived signals, autocrine signaling in neurons involving neurotrophins and other secreted signaling molecules has been shown to affect neuronal survival, axon growth, and other aspects of neuronal development and function (Wright et al., 1992, Acheson et al., 1995, O’Keeffe et al., 2008, Cheng et al., 2011, Ryu et al., 2013). However, neuronal autocrine signaling is difficult to reconcile with neurotrophic theory because it is not clear how autonomous signaling loops in neurons could contribute to the establishment of distinctive patterns of tissue innervation.

From a PCR screen to identify novel regulators of neuronal survival and axon growth, we detected expression of transcripts encoding CD40L (TNFSF5), a member of the tumor necrosis factor superfamily (TNFSF), and CD40 (TNFRSF5), a member of the TNF receptor superfamily (TNFRSF), in the experimentally tractable sympathetic neurons of the mouse superior cervical ganglion (SCG) at the stage when the axons of these neurons are ramifying extensively in their target tissues. CD40L and CD40 are prominently expressed in the immune system, where they play a central role in the generation of immune responses and the pathogenesis of autoimmune disease (Calderhead et al., 2000, Peters et al., 2009). Whereas there is some evidence for the appearance of a neurodegenerative phenotype in aged CD40 knockout mice (Tan et al., 2002), CD40 and CD40L are not known to play any role in neural development. We demonstrate that CD40 autocrine signaling enhances NGF-promoted axonal growth and branching, is regulated by the level of NGF in targets, and exerts regional effects on innervation density in vivo. These findings resolve the long-standing conundrum of neuronal autocrine signaling by uncovering a mechanism of differential regulation of autocrine signaling within neuronal populations, resulting in specific regional effects on tissue innervation.

## Results

### CD40 and CD40L Are Co-expressed in Perinatal SCG Neurons

**Figure 1:**
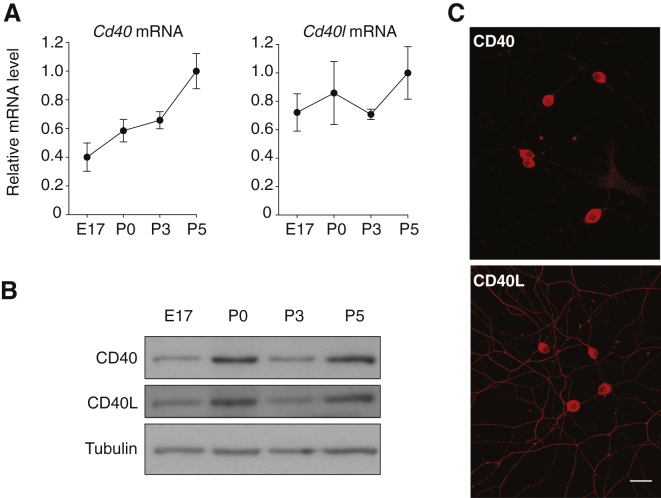
SCG Neurons Express CD40 and CD40L (A) Levels of *Cd40* and *Cd40l* mRNA relative to reference mRNAs in SCG of different ages. The data are normalized to a value of 1.0 at the peak of expression at P5. Mean ± SEM of data from three separate sets of ganglia at each age is shown. (B) Representative western blot of lysates of SCG of different ages. (C) Images of representative P3 SCG neurons cultured for 24 hr in medium containing 1 ng/ml NGF labeled with either anti-CD40 or anti-CD40L. The scale bar represents 50 μm.

Quantitative PCR revealed the expression of *Cd40* and *Cd40l* transcripts in the SCG of late fetal and early postnatal mice during the stage when sympathetic axons are ramifying extensively in their targets (Figure 1A). Compared with adult spleen, where CD40 and CD40L are expressed at very high levels, the levels of *Cd40* mRNA and *Cd40l* mRNA were 8.8-fold and 950-fold lower, respectively, in the SCG at P5. Western blotting confirmed the presence of CD40 and CD40L protein in the developing SCG (Figure 1B). In dissociated SCG cultures, the great majority of neurons, positively identified by βIII-tubulin labeling, were labeled by anti-CD40 (91.5 ± 2.4; mean ± SEM) and by anti-CD40L (87.4 ± 2.0; Figure 1C). The small number of non-neuronal cells in these cultures exhibited a very low level of CD40 and CD40L immunostaining. No neurons were labeled when primary antibodies were omitted, and anti-CD40 did not label neurons cultured from *Cd40*^−/−^ mice. These observations suggest that the great majority of sympathetic neurons co-express CD40 and CD40L when sympathetic axons are innervating their targets.

### Autocrine CD40 Reverse Signaling Enhances NGF-Promoted Axon Growth

**Figure 2:**
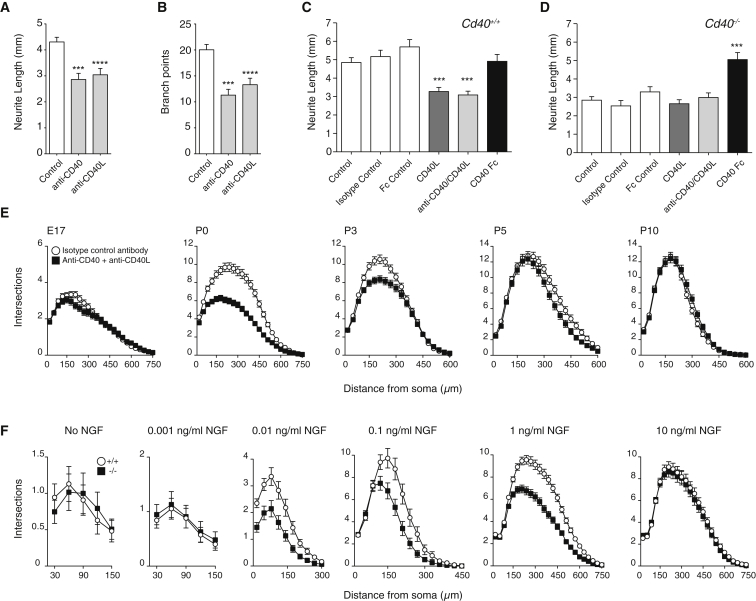
CD40 Signaling Enhances NGF-Promoted Neurite Growth (A and B) Neurite arbor length (A) and branch point number (B) of P3 SCG neurons cultured for 24 hr with 1 ng/ml NGF plus function-blocking anti-CD40, function-blocking anti-CD40L, or isotype control antibodies (2 μg/ml). (C) Neurite arbor length of P3 SCG neurons cultured for 24 hr in media supplemented with 1 ng/ml NGF alone (control) or 1 ng/ml NGF plus isotype control antibodies (Iso control), Fc fragment (Fc control), function-blocking anti-CD40 and anti-CD40L (FB antibodies), 1 μg/ml CD40L, or 1 μg/ml CD40-Fc. (D) Neurite arbor length of SCG neurons obtained from P3 *Cd40*^−/−^ littermates cultured under the same conditions as in (C). ^∗∗∗∗^p < 0.0001; ^∗∗∗^p < 0.001, ANOVA with *Bonferroni* correction, statistical comparison with controls. (E) Sholl plots of neurite arbors of E17–P10 SCG neurons cultured for 24 hr with 1 ng/ml NGF plus either function blocking or isotype control antibodies. (F) Sholl plots of the neurite arbors of P3 SCG neurons of *Cd40*^+/+^ and *Cd40*^−/−^ littermates grown for 24 hr with a concentration range of NGF and 25 μM Boc-D-FMK to prevent apoptosis at low NGF concentrations. Mean ± SEM data of >150 neurons per condition combined from three to five experiments of each type.

The co-expression of CD40L and CD40 raised the possibility of autocrine signaling. To test this possibility and to ascertain how autocrine signaling might influence SCG neuron development, we studied the effects of blocking the interaction of CD40L and CD40 using function-blocking antibodies and quantified neuronal survival and neurite growth. Whereas NGF-promoted neuronal survival was unaffected by these antibodies (Figure S1A), neurite arbor size and complexity were markedly reduced. In the presence of NGF, each function-blocking antibody caused highly significant reductions in total length (Figure 2A) and branch point number (Figure 2B) compared with isotype control antibodies. Representative images of neurons grown under these conditions are shown in Figure S1B. Because these data were obtained from non-contiguous neurons cultured at exceptionally low density, these findings suggest that CD40 autocrine signaling enhances NGF-promoted neurite growth but does not affect NGF-promoted survival.

Interaction of CD40 and membrane-integrated CD40L can initiate bi-directional signaling: CD40-mediated forward signaling and CD40L-mediated reverse signaling (Sun and Fink, 2007). To determine whether CD40/CD40L interaction enhances NGF-promoted axon growth by either forward or reverse signaling, we ascertained whether either soluble CD40L or soluble CD40-Fc chimera (in which the extracellular domain of CD40 is linked to the Fc part of human IgG1) interferes with NGF-promoted axon growth. Whereas soluble CD40L reduced NGF-promoted axon growth to the same extent as the function-blocking anti-CD40 and anti-CD40L antibodies, CD40-Fc had no significant effect on NGF-promoted axon growth (Figure 2C). The most parsimonious explanation for these results is that the added soluble CD40L competes with endogenous membrane-integrated CD40L for binding to endogenous CD40 and thereby blocks endogenous CD40L-mediated reverse signaling. Further support for reverse signaling came from the phenotype rescue experiments on SCG neurons obtained from *Cd40*−/− mice. The extent of NGF-promoted axon growth from these neurons was similar to that of SCG neurons of wild-type littermates treated with function-blocking anti-CD40 and anti-CD40L antibodies (Figure 2D). CD40-Fc fully restored the extent of NGF-promoted axon growth from CD40-deficient neurons to that of wild-type neurons, suggesting that soluble, rather than membrane-integrated CD40, is sufficient to rescue the impaired axon growth phenotype of these neurons. The observation that neither soluble CD40L nor function-blocking antibodies reduced the extent of NGF-promoted axon growth from CD40-deficient neurons (Figure 2D) additionally shows that these reagents do not exert non-specific suppressive effects on axon growth.

### CD40 Signaling Enhances NGF-Promoted Axon Growth during a Perinatal Window

To ascertain whether CD40 autocrine signaling reduces NGF-promoted neurite growth during a particular period of sympathetic neuron development, we examined the effect of function-blocking anti-CD40L and anti-CD40 in cultures established over a range of ages. Sholl analysis showed that these antibodies decreased NGF-promoted neurite growth most markedly in P0 and P3 SCG cultures (Figure 2E). Quantification of neurite length and branching revealed consistent decreases at E17 and P5, but these decreases did not reach significance (p > 0.05; t tests; n = 3 experiments at each age). Highly significant decreases in neurite length and branching were observed in P0 and P3 cultures (p < 0.0001 for length and branching at P0; p < 0.05 for length and p < 0.0001 for branching at P3; unpaired two-tailed t test; n = 3 experiments at each age). No consistent effect of the antibodies on neurite length and branching were observed in P10 cultures. These findings suggest that CD40L autocrine signaling enhances NGF-promoted neurite growth from SCG neurons maximally during the period of development when SCG axons are ramifying extensively in their target tissues under the influence of target-derived NGF (Davies, 2009).

### CD40 Signaling Enhances Axon Growth Promoted by Low Concentrations of NGF

To provide a more stringent test of the effect of CD40 signaling on NGF-promoted neurite growth, we compared the size and complexity of the neurite arbors of SCG neurons obtained from neonatal CD40-deficient mice and wild-type littermates. Detailed dose-response analysis revealed that absence of CD40 reduced NGF-promoted neurite growth over a restricted range of low NGF concentrations. In cultures containing 0.01, 0.1, and 1 ng/ml NGF, the neurite arbors of CD40-deficient neurons were markedly smaller and less complex than those of wild-type mice, whereas at lower and higher concentrations of NGF, there were no significant differences in neurite arbor size and complexity (Figure 2F). All cultures received the broad-spectrum caspase inhibitor Boc-D-FMK to prevent apoptosis at low levels of NGF. The great majority of neurons survived in these experiments, and there were no significant differences in survival between the experimental groups (not shown). These findings suggest that CD40 signaling does not affect neurite growth on its own but enhances NGF-promoted neurite growth and branching over a narrow range of low NGF concentrations.

### NGF Negatively Regulates Expression of CD40L and CD40

**Figure 3:**
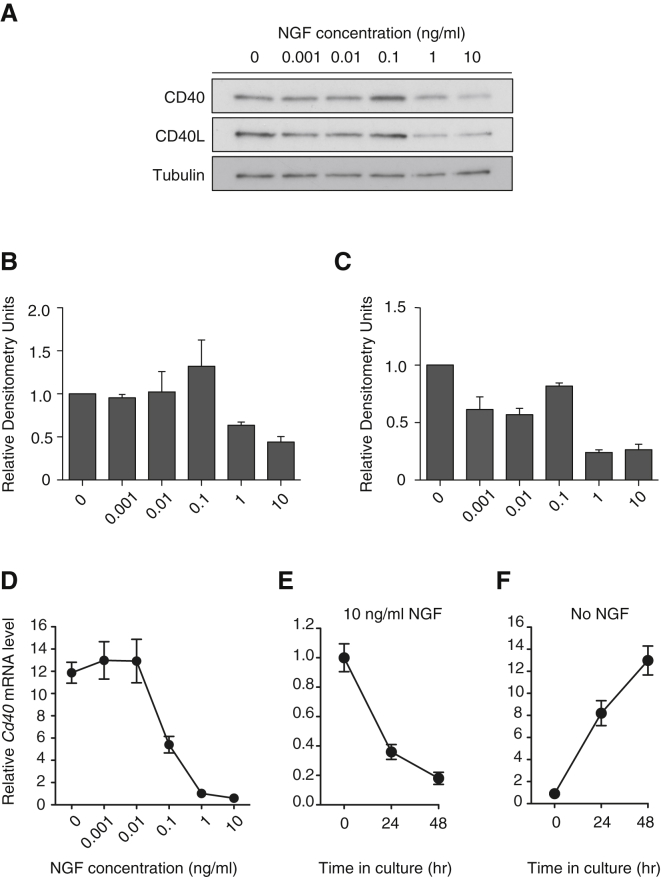
Negative Regulation of CD40 Expression by NGF (A) Representative western blots of P3 SCG neuron lysates probed for CD40 and CD40L after 48 hr culture with different levels of NGF. All cultures received 25 μM Boc-D-FMK. (B) Densitometry for CD40 from multiple western blots. (C) Densitometry for CD40L from multiple western blots. (D–F) Level of *Cd40* mRNA in P3 SCG cultures grown with a range of NGF concentrations for 48 hr (D) or for 24 and 48 hr with 10 ng/ml NGF (E) or without NGF (F). Data normalized to 1.0 at plating (n = 3 experiments). Mean ± SEM data combined from three to five experiments of each type.

The lack of effect of *Cd40* deletion on neurite growth at high NGF concentrations could be due to negative regulation of the expression of CD40L and/or CD40 by NGF. To test this possibility, SCG neurons were cultured with a range of NGF concentrations and western blotting was used to assess the relative levels of CD40L and CD40 after 48 hr. This analysis revealed a clear decrease in the levels of both proteins with increasing levels of NGF (Figures 3A–3C). Because the levels of *Cd40* mRNA are much higher than those of *Cd40l* mRNA and can be easily and accurately quantified by qPCR in low-density SCG cultures, we carried further detailed analysis of the influence of NGF on *Cd40* mRNA expression. There was a very clear inverse relationship between the levels of NGF and *Cd40* mRNA, with a very marked decrease in *Cd40* mRNA at NGF concentrations between 0.01 and 1 ng/ml (Figure 3D). To ascertain whether these marked differences in *Cd40* mRNA observed at 48 hr were due to increases and/or decreases in *Cd40* mRNA with time in culture, we measured the levels of *Cd40* mRNA in freshly dissociated SCG neurons and in SCG neurons cultured for 24 and 48 hr with and without 10 ng/ml NGF. In cultures containing NGF, there was an ∼60% decrease in the level of *Cd40* mRNA relative to the level in freshly dissociated SCG neurons after 24 hr in culture and a further decrease by 48 hr (Figure 3E). In contrast, there was a marked ∼8-fold increase in the level of *Cd40* mRNA during the first 24 hr in cultures lacking NGF and a further increase by 48 hr (Figure 3F). These data indicate that *Cd40* mRNA expression increases in the absence of NGF and is decreased by relatively high levels of NGF.

### Sympathetic Innervation of Tissue Expressing Low, but Not High, Levels of NGF Is Reduced in Mice Lacking CD40

**Figure 4:**
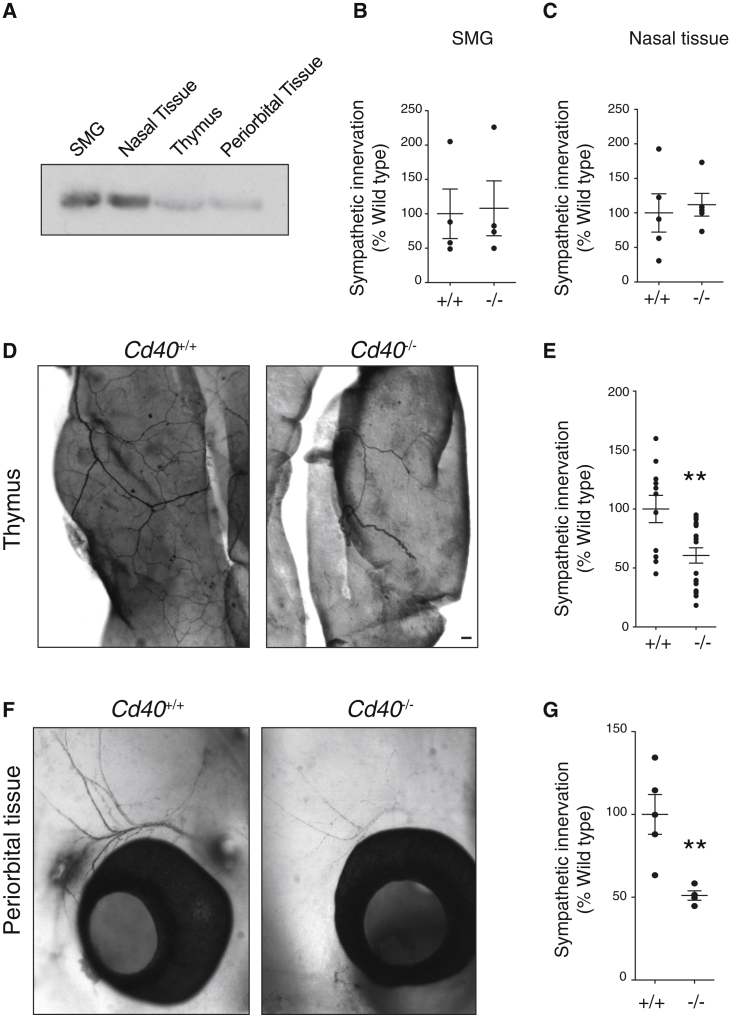
Reduced Sympathetic Innervation of Low-NGF-Expressing, but Not High-NGF-Expressing, Tissue in CD40-Deficient Mice (A) Representative western blot of P3 submandibular salivary gland (SMG), P3 nasal tissue, P3 thymus, and E16.5 periorbital cutaneous tissue probed for NGF, showing the band corresponding to mature NGF. (B and C) Scatterplots of sympathetic innervation density in P3 SMG (B) and P3 nasal turbinate tissue (C). (D and E) Representative images of P3 thymus whole mounts of *Cd40*^+/+^ and *Cd40*^−/−^ mice stained for TH-positive sympathetic fibers (D) and scatterplots of innervation density (E). (F and G) Representative images of whole mounts of E16.5 periorbital cutaneous tissue of *Cd40*^+/+^ and *Cd40*^−/−^ mice stained for TH-positive sympathetic fibers (F) and scatterplots of innervation density (G). Mean ± SEM. ^∗∗^p < 0.01; t test. The scale bars represent 100 μm.

To assess the physiological relevance of our in vitro findings, we studied the sympathetic innervation of several tissues that express markedly different levels of NGF. Western analysis showed that the densely innervated submandibular salivary gland and nasal turbinate tissue express much higher levels of mature NGF than the sparsely innervated thymus and periorbital cutaneous tissue (Figure 4A). These differences in NGF level accord with earlier studies that reported relatively high levels of NGF and *Ngf* mRNA in the submandibular salivary gland compared with the thymus (Shelton and Reichardt, 1984, Heumann et al., 1984).

We assessed sympathetic innervation density by quantifying immunostaining for tyrosine hydroxylase, a rate-limiting enzyme for noradrenaline synthesis specifically expressed in sympathetic fibers. Innervation density in the densely innervated submandibular salivary gland and nasal turbinate tissue was assessed by quantifying tyrosine hydroxylase staining in histological sections (Kisiswa et al., 2013). Because there are so few sympathetic fibers in the thymus and periorbital cutaneous tissue, we assessed innervation density by quantifying tyrosine-hydroxylase-positive fibers in cleared whole-mount tissue preparations (Kisiswa et al., 2013). Quantification of sympathetic innervation density in the submandibular salivary gland, nasal turbinate tissue, and thymus was carried out at P3 when sympathetic innervation is well established. However, in order to visualize cutaneous sympathetic fibers in cleared whole-mount preparations adequately, it was necessary to examine this tissue in late fetal mice at E16.5 (Glebova and Ginty, 2004). Quantification of sympathetic innervation density in the high-NGF-expressing submandibular salivary gland and nasal turbinate tissue revealed no significant differences between *Cd40*^+/+^ and *Cd40*^−/−^ mice (Figures 4B and 4C). Representative tyrosine-hydroxylase-stained sections of these tissues are shown in Figures S2A and S2B. In contrast, there were highly significant reductions in the sympathetic innervation density of the thymus and periorbital cutaneous tissue of *Cd40*^−/−^ mice compared with *Cd40*^+/+^ mice (Figures 4D–4G). These findings suggest that CD40 signaling selectively enhances the sympathetic innervation of low-NGF-expressing, but not high-NGF-expressing, tissue.

## Discussion

We have uncovered an autocrine-signaling loop in developing sympathetic neurons involving CD40 and CD40L that enhances NGF-promoted neurite growth but has no effect on NGF-promoted survival. This autocrine-signaling loop has no effect on neurite growth on its own and only enhances neurite growth from neurons cultured with NGF over a low concentration range. The reduction of NGF-promoted axon growth by soluble CD40L, but not by CD40-Fc, together with the rescue of the reduced axon growth phenotype of CD40-deficient neurons by CD40-Fc suggests that enhancement of NGF-promoted axon growth by the CD40/CD40L autocrine-signaling loop occurs by CD40L-mediated reverse signaling. In addition to CD40L, several other members of the TNFSF, including TNF, FasL, LIGHT, GITRL, and APRIL, have been shown to either enhance or repress axon growth from a variety of developing neurons (Desbarats et al., 2003, Gutierrez et al., 2008, Gutierrez et al., 2013, Gavaldà et al., 2009, McKelvey et al., 2012, Kisiswa et al., 2013, Osório et al., 2014, Wheeler et al., 2014). Of these, only TNF has been shown to affect axon growth by reverse signaling (Kisiswa et al., 2013, Wheeler et al., 2014). Our findings not only extend the importance of the TNFSF in regulating axon growth in neural development but show that reverse signaling is not restricted to TNF in the nervous system.

The negative regulation of CD40 and CD40L expression by NGF explains why the effects of CD40/CD40L autocrine signaling on axon growth are curtailed at higher NGF concentrations. The physiological relevance of our in vitro observations was confirmed by quantification of sympathetic innervation density of tissues that express different levels of NGF. In CD40-deficient mice, the sympathetic innervation density of the low-NGF-expressing thymus and periorbital cutaneous tissue was significantly reduced compared with wild-type littermates, whereas the sympathetic innervation density of the high-NGF-expressing submandibular salivary gland and nasal turbinate tissue was unaffected. The growth and branching of sympathetic fibers in at least two of these tissues, the periorbital cutaneous tissue and the submandibular salivary gland, has been shown to be critically dependent on NGF in vivo (Glebova and Ginty, 2004). Our findings therefore suggest that CD40 signaling selectively modulates NGF-dependent innervation in vivo and reveal a fundamentally novel mechanism for adjusting the innervation density of specific tissues based on the differential regulation of an autocrine-signaling loop in the innervating population of neurons in accordance with the level of NGF in the tissue they innervate. Because some members of the TNFSF, such as GITRL and APRIL, appear to act by an autocrine mechanism, our current findings raise the possibility that analogous paradigms might operate in regulating tissue innervation by these cytokines.

Why has such a mechanism evolved? Because NGF promotes both survival and axon growth in the developing peripheral nervous system, it might not be possible to attain the physiologically appropriate innervation density of particular tissues by a limited number of neurons if there was an invariant relationship between the survival-promoting and axon-growth-promoting effects of NGF. For example, whereas a low level of NGF in certain tissues may be sufficient to support the required number of innervating neurons, this may be inadequate to promote enough axon growth and branching for optimal sympathetic function. The CD40 autocrine-signaling loop breaks an otherwise invariant relationship between survival and axon growth, facilitating physiologically appropriate innervation density by a limited number of neurons in low-NGF-expressing tissue. In future work, it will be informative to ascertain how CD40L-mediated reverse signaling influences downstream NGF signaling to modulate axon growth selectively and how extensively this and analogous paradigms of autocrine signaling operate in the nervous system.

## Experimental Procedures

### Primary Neuron Culture

SCGs were dissected from mice that were bred and housed in accordance with guidelines approved by the Cardiff University Ethical Review Board and the Home Office Animals (Scientific Procedures) Act, 1986. SCG neurons were cultured at very low density on poly-ornithine- and laminin-coated 4-well tissue culture dishes in defined medium. Neurite arbors were labeled by the fluorescent vital dye calcein-AM after 24 hr, and the images were analyzed to obtain neurite length, branch point number, and Sholl profiles (Gutierrez and Davies, 2007).

### Quantitative PCR

The levels of *Cd40l* and *Cd40* mRNAs were quantified by real-time PCR relative to a geometric mean of mRNAs for house-keeping enzymes. See Supplemental Information for primer details and reaction conditions.

### Immunohistochemistry and Immunocytochemistry

Tissue and cultures were fixed in 4% paraformaldehyde in 0.12 M phosphate buffer. Frozen sections were cut at 14 μm, and permeabilized cultures were incubated for 18 hr at 4°C with primary antibody (anti-tyrosine hydroxylase, anti-CD40, or anti-CD40L). After washing, the tissue and cultures were incubated for 1 hr with donkey anti-rabbit Alexa 488 antibody, washed, and imaged by confocal microscopy.

### Immunoblotting

Tissue and neuron cultures were lysed in RIPA lysis buffer containing protease and phosphatase inhibitors. After removal of insoluble material by centrifugation, protein concentration was determined by Bradford assay and equal quantities of protein were separated on 10% SDS-PAGE gels and were transferred to PVDF membranes. After blocking, the membranes were incubated with primary antibodies overnight at 4°C. After washing, the membranes were incubated with HRP-conjugated secondary antibodies and the blots were developed by chemiluminescence.

### Whole-Mount Preparations

Paraformaldehyde fixed tissue was processed to label tyrosine-hydroxylase-positive sympathetic fibers by DAB-HRP staining followed by clearing in benzyl alcohol:benzyl benzoate as described previously (Kisiswa et al., 2013) with modifications (Supplemental Information).

### Quantification of Sympathetic Innervation Density

Tissue from *Cd40*^*+/+*^ and *Cd40*^*−/−*^ littermates was processed at the same time, and quantification was done blind. Sympathetic fiber density in the submandibular gland and nasal turbinate tissue was carried out on TH-labeled images of every fifth section through the tissue using NIH-ImageJ to estimate TH-positive fibers per unit area. Sympathetic fiber density in the thymus and periorbital cutaneous tissue was analyzed in TH-labeled whole-mount preparations. TH-positive fibers per unit image area were estimated from manually traced fibers in Adobe Photoshop images of the tissue.

## Author Contributions

T.G.M. and L.H. did the cell culture, T.G.M. quantified tissue innervation, L.H. undertook the western analysis, S.W. carried out the qPCR, A.M.D. and S.W. supervised the project, and A.M.D. wrote the manuscript.

## Supplemental Information

Document S1. Supplemental Experimental Procedures and Figures S1 and S2

Document S2. Article plus Supplemental Information
