# Supplementary material for: Regulation of Autocrine Signaling in Subsets of Sympathetic Neurons Has Regional Effects on Tissue Innervation
Source: Cell Rep. 2015 Mar 5;10(9):1443–9. doi: 10.1016/j.celrep.2015.02.016 (PMC4407286; doi:10.1016/j.celrep.2015.02.016)
Supplement: Document S1. Supplemental Experimental Procedures and Figures S1 and S2 [file mmc1.pdf]

Cell Reports

Supplemental Information

Regulation of Autocrine Signaling in Subsets of Sympathetic Neurons Has Regional Effects on Tissue Innervation

Thomas G. McWilliams, Laura Howard, Sean Wyatt, and Alun M. Davies

Supplemental Experimental Procedures

Primary neuron culture

SCG were trypsinized and plated at very low density (~ 50 neurons per well) in poly-ornithine and laminin-coated 4-well tissue culture dishes (Greiner, Gloucestershire, UK) in serum-free Hams F14 medium (Kisiswa et al., 2013) supplemented with 0.25% Albumax I (Invitrogen, Paisley, UK). The majority of cultures were established from wild-type CD1 mice. *Cd40* null mutant mice in a C57BL6/J background were purchased from The Jackson Laboratory (Maine, USA). All animal experiments were conducted in accordance with the 1986 Animal Procedures Act, Home Office (UK). Mice of both sexes were used.

Neuronal survival was estimated by counting the number of attached neurons within each well 2 hours after plating and again after 24 and 48 hours. Analysis of the size and complexity of neurite arbors was carried out 24 hours after plating. At this time, the neurite arbors were labeled by incubating the neurons with the fluorescent vital dye calcein-AM (1:1000, Invitrogen, Paisley, UK). Images of neurite arbors were analyzed to obtain neurite length, branch point number and Sholl profiles (Gutierrez and Davies, 2007).

Recombinant NGF was obtained from R&D Systems (Minneapolis, USA). Function blocking antibodies to CD40 and CD40L were obtained from BD Pharmingen (Oxford, UK, catalogue number 553721) and Abcam (Cambridge, UK, catalogue number ab99895), respectively, and the corresponding isotype control antibodies were obtained from BD Pharmingen (catalogue number 553957) and Abcam (catalogue number ab18426). CD40L, CD40-Fc and Control Fc were obtained from Enzo, catalogue numbers ALX-522-120-C010, ALX-522-016 and ALX-203-004.

Quantitative PCR

The levels of *Cd40l* and *Cd40* mRNAs were quantified by real-time PCR relative to a geometric mean of mRNAs for the house keeping enzymes glyceraldehyde phosphate dehydrogenase (*Gapdh*), succinate dehydrogenase (*Sdha*) and hypoxanthine phosphoribosyltransferase-1 (*Hprt1*). Total RNA was extracted from dissected SCG or cultured neurons using the RNeasy Micro extraction kit (Qiagen, Crawely, UK) and 5 µl was reverse transcribed for 1 h at 45°C using the AffinityScript kit (Agilent, Berkshire, UK) in a 25 μl reaction according to the manufacturer's instructions. 2 μl of cDNA was amplified in a 20 μl reaction volume using Brilliant III ultrafast qPCR master mix reagents (Agilent). PCR products were detected using dual-labeled (FAM/BHQ1) hybridization probes specific to each of the cDNAs (MWG/Eurofins, Ebersberg, Germany). The PCR primers were: *Cd40l* forward: 5'-TGG ATC TGA GAG AAT CTT ACT-3' and reverse: 5'-AGT CAC GTT GAC AAA CAC-3' ; *Cd40* forward: 5′-CTT TGG AGT TAT GGA GAT G - 3′ and reverse: 5′-ATG ACT GAT TGG AGA AGA-3′; *Gapdh* forward: 5′-GAG AAA CCT GCC AAG TAT G-3′ and reverse: 5′-GGA GTT GCT GTT GAA GTC-3′; *Sdha* forward: 5′-GGA ACA CTC CAA AAA CAG-3′ and reverse: 5′-CCA CAG CAT CAA ATT CAT-3′; *Hprt1* forward: TTA AGC AGT ACA GCC CCA AAA TG and reverse: AAG TCT GGC CTG TAT CCA ACA C. Dual-labeled probes were: *Cd40l*: 5'-FAM-CGG CAA ATA CCC ACA GTT CCT-BHQ1-3'; *Cd40*: 5'-FAM-CCA CTG AGA CCA CTG ATA CCG-BHQ1-3'; *Gapdh*: 5′-FAM-AGA CAA CCT GGT CCT CAG TGT-BHQ1-3; *Sdha*: 5′-FAM-CCT GCG GCT TTC ACT TCT CT-BHQ1-3, *Hrpt1*: FAM-TCG AGA GGT CCT TTT CAC CAG CAA G-BHQ1. Forward and reverse primers were used at a concentration of 150 nM and dual-labeled probes were used at a concentration of 300 nM. PCR was performed

using the Mx3000P platform (Agilent) using the following conditions: 45 cycles of 95°C for 12 s and 60°C for 35 seconds. Standard curves were generated for each cDNA for every real time PCR run, by using serial threefold dilutions of reverse transcribed mouse adult brain total RNA (Zyagen, San Diego, USA). Relative mRNA levels were quantified in four separate sets of dissected tissues and cultured cells for each experiment. Primer and probe sequences were designed using Beacon Designer software (Premier Biosoft, Palo Alto, USA).

Immunohistochemistry and immunocytochemistry

For immunohistochemistry, tissues were fixed in fresh 4% paraformaldehyde in 0.12 M phosphate buffer, pH 7.2 for 3 h at 4°C. After washing in PBS, the tissue was cryoprotected in 30% sucrose before being frozen. OCT embedded tissues were frozen in isopentene cooled with dry ice and were serially sectioned at 14 μm. The sections were mounted on electrostatically charged slides (Xtra-adhesive slides, Surgipath, Leica Microsystems, Peterborough, UK), blocked with 5% BSA and 0.2% Triton X-100 (Sigma-Aldrich, Dorset, UK) in PBS for 1 h at room temperature, and then incubated for 18 h at 4°C with anti-tyrosine hydroxylase (1:200, Millipore, Dundee, UK, catalogue number AB152) in PBS with 1% BSA. The sections were washed in PBS and were incubated for 1 hr with donkey anti-rabbit 488 Alexa Fluor secondary antibody (Life Technologies, UK, catalogue number A-21206). After washing with PBS, the sections were mounted with VECTASHIELD® Hard Set Mounting Media (Vector Laboratories, Cambridgeshire, UK).

For immunocytochemistry, cultures were fixed as above for 15 min at room temperature and were washed extensively with PBS before permeablisation and blocking of nonspecific binding for 1 h at room temperature as above. The cultures were incubated overnight at 4°C with primary antibody in PBS containing 1% BSA. The following primary antibodies were used:

rabbit polyclonal anti-CD40L (Abcam, Cambridge, UK, 1/200, catalogue number ab2391) and rabbit polyclonal anti-CD40 (Abcam, Cambridge, UK, 1/200, catalogue number ab13545). After washing with PBS, the cultures were incubated with Alexa Fluor conjugated donkey anti-rabbit secondary antibody. Images were obtained using a Zeiss LSM510 confocal microscope.

Immunoblotting

NGF was quantified in dissected submandibular salivary glands, nasal turbinate tissue, thymuses and periorbital tissue cutaneous with the most superficical level of the skin removed by tungsten needles, since sympathetic fibers are restricted to the subcutaneous and dermal levels. CD40 and CD40L were quantified in tissues and neurons cultured at high density. The tissue and neurons were lysed in ice-cold RIPA lysis buffer supplemented with protease and phosphatase inhibitor cocktail mix (Sigma) and insoluble debris was removed by centrifugation. Protein concentration was determined by Bradford assay (BioRad, Hertfordshire, UK), using BSA standards on a Tecan Infinite Plate Reader. Unless stated otherwise, equal quantities of protein were separated on 10% SDS-PAGE gels and were transferred to PVDF membranes (Immobilon-P, Millipore) using the BioRad TransBlot Apparatus. After blocking for 1 hr at RT with 5% skimmed milk in PBS containing 0.1% Tween-20 (PBST), membranes were incubated in 1% block solution containing primary antibodies overnight at 4°C with gentle agitation. Membranes were washed extensively in PBST before incubation for 1 hr at room temperature with the appropriate species specific HRP-conjugated secondary antibodies (1:2000, Promega, Southampton, UK) in 1% block solution. Blots were developed by chemiluminescence using Amersham Hyperfilm ECL (GE Life Sciences, Buckinghamshire, UK) and Immunocruz Luminol Reagents (Santa Cruz Biotechnology, CA, USA). The following primary antibodies were used: rabbit polyclonal anti-CD40L (Abcam, Cambridge, UK, 1:500, catalogue number

ab65854), rabbit polyclonal anti-CD40 (Abcam, Cambridge, UK, 1:1000, catalogue number ab13545), rabbit polyclonal anti-NGF (Santa Cruz, CA, USA, 1:1000, catalogue number sc-548) and β-III tubulin (R&D systems, Minneapolis, USA, 1:5000, catalogue number MAB1195).

Whole-mount preparations

Thymuses of P3 *Cd40*$^{+/+}$ and *Cd40*$^{-/-}$ pups and heads of E16.5 *Cd40*$^{+/+}$ and *Cd40*$^{-/-}$ embryos for were fixed in 4% paraformaldehyde for 24 h. The tissue was serially dehydrated in methanol and endogenous peroxidase activity was quenched overnight at 4°C in a solution of 80% methanol and 20% DMSO containing 3% H_2O_2. The tissue was rehydrated in 50% methanol in PBS for 1 h, 30% methanol in PBS for 1 h, and PBS for 1 h, and was blocked overnight at 4 °C with PBS containing 4% BSA and 1% Triton. The tissue was incubated with rabbit polyclonal anti-tyrosine hydroxylase (1:200, Millipore, Dundee, UK, catalogue number AB152) in 1% BSA plus 1% Triton X-100 in PBS for 72 h at 4°C. After washing three times for 2 h in 1% Triton X-100 in PBS at room temperature, the tissue was kept at 4°C overnight before incubating with HRP-conjugated anti-rabbit secondary antibody (1:300, Promega) in 1% BSA plus 1% Triton X-100 at 4 °C overnight. The tissue was washed with PBS containing 1% Triton X-100 for 2 h at room temperature. Tyrosine hydroxylase-positive fibers were visualized by DAB-HRP staining. After a brief post-fixation in 4% paraformaldehyde at room temperature, the tissue was serially dehydrated in solutions of 50% methanol/PBS for 2 h, 80% methanol/PBS for 2h and 100% methanol overnight at 4°C. Prior to imaging, dehydrated tissues were cleared in a solution of (1:2) benzyl alcohol:benzyl benzoate.

Quantification of sympathetic innervation density

Batches of tissue from *Cd40*$^{+/+}$ and *Cd40*$^{-/-}$ littermates were processed at the same time and quantification was done blind. For the submandibular gland and nasal turbinate tissue, images of every 5th section were acquired and converted to grey scale in NIH-ImageJ with a Gaussian blur correction (ʃ = 1) applied to each image. Multiple random regions in these images were analyzed in which the ratio of immunoreactive TH–positive fibers to total area of the region was estimated. To ensure consistent analysis across all conditions, multiple images from all mice were initially analyzed to generate a uniform threshold value, which was applied to every image analyzed. An average of TH-positive fibres per section area analyzed was obtained for each animal (approximately 30 sections per SMG or nasal turbinate tissue analyzed per animal). The data are expressed as a percentage of the mean wild type data. For the thymus and periorbital cutaneous tissue, TH-positive fibers were traced manually in images of the whole thymus or periorbital tissue in Adobe Photoshop CS5.1. A uniform threshold value was applied to all images in NIH-ImageJ to generate values for pixel intensity of traced fibers per area for the thymus and periorbital cutaneous tissue. The data are expressed as a percentage of the mean of the wild type data.

Supplemental Figure Legends

Figure S1. Influence of function-blocking antibodies on neuronal survival and axon growth in culture, Related to Figure 2. (**A**) Percentage survival of P3 SCG neurons cultured for 48 hours with 1 ng/ml NGF plus either function blocking anti-CD40 and anti-CD40L antibodies or isotype control antibodies (2 μg/ml). There are no significant differences between the experimental groups. (**B**) Representative images of P3 SCG neurons after 24 hours incubation with 1 ng/ml NGF plus either function blocking anti-CD40 and function blocking anti-CD40L or isotype control antibodies (2 μg/ml). Scale bar, 100 μm.

Figure S2. No reduction in the innervation density of high NGF expressing tissues, Related to Figure 4. (**A**) Cryosections of nasal turbinate tissue of P3 *Cd40*$^{+/+}$ and *Cd40*$^{-/-}$ mice stained for tyrosine hydroxylase-positive sympathetic fibers. (**B**) Cryosections of submandibular salivary gland of P3 *Cd40*$^{+/+}$ and *Cd40*$^{-/-}$ mice stained for tyrosine hydroxylase-positive sympathetic fibers. Scale bars, 100 μm.

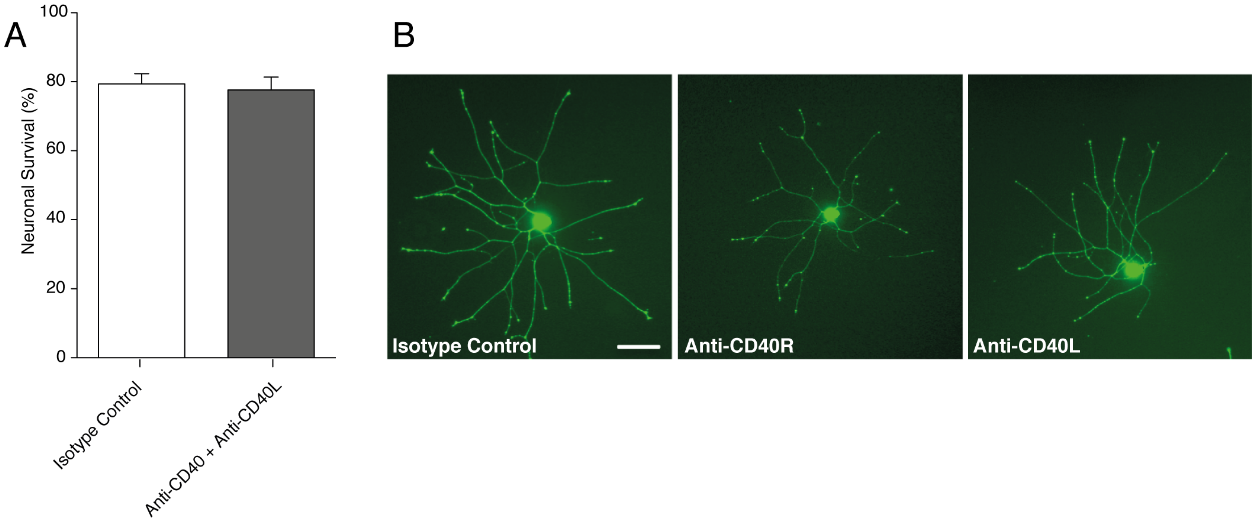
A
100
80
60
40
20
0
Neuronal Survival (%)
Isotype Control
Anti-CD40 + Anti-CD40L
B
Isotype Control
Anti-CD40R
Anti-CD40L

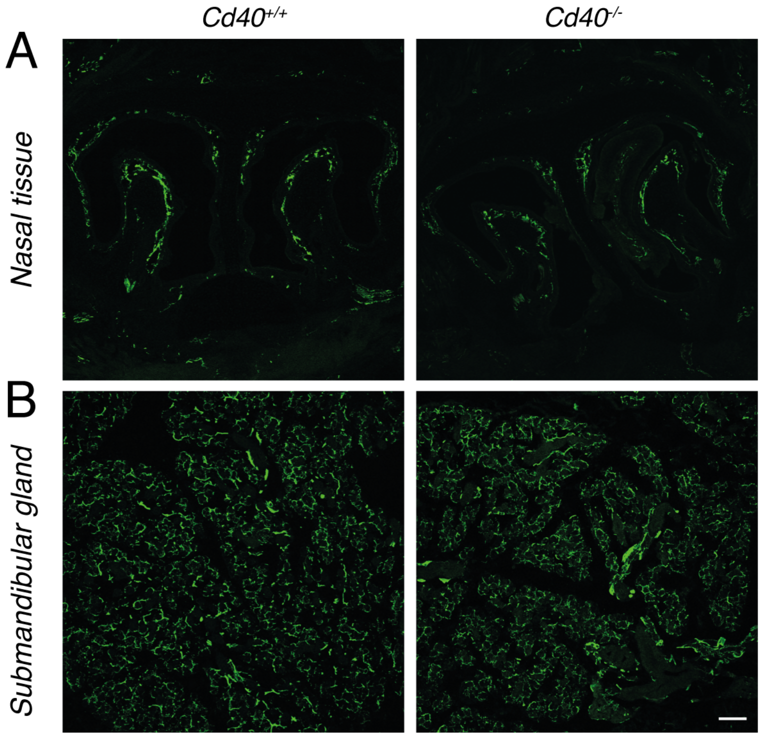
Cd40+/+
Cd40-/-
A
Nasal tissue
B
Submandibular gland
